# Iron Overload and Abdominal Aortic Aneurysm

**DOI:** 10.31083/j.rcm2510361

**Published:** 2024-10-09

**Authors:** Yunyi Li, Quan Zhou, Kai Zhang, Xiao Meng

**Affiliations:** ^1^The Key Laboratory of Cardiovascular Remodeling and Function Research, Chinese Ministry of Education, Chinese National Health Commission and Chinese Academy of Medical Sciences, The State and Shandong Province Joint Key Laboratory of Translational Cardiovascular Medicine, Department of Cardiology, Qilu Hospital, Cheeloo College of Medicine, Shandong University, 250012 Jinan, Shandong, China

**Keywords:** iron overload, abdominal aortic aneurysm, inflammation, oxidative stress, endothelial function

## Abstract

Abdominal aortic aneurysm (AAA) is a chronic vascular degenerative disease characterized by progressive segmental dilation of the abdominal aorta. The rupture of an AAA represents a leading cause of death in cardiovascular diseases. Despite numerous experimental and clinical studies examining potential drug targets and therapies, currently there are no pharmaceutical treatment to prevent AAA growth and rupture. Iron is an essential element in almost all living organisms and has important biological functions. Epidemiological studies have indicated that both iron deficiency and overload are associated with adverse clinical outcomes, particularly an increased risk of cardiovascular events. Recent evidence indicates that iron overload is involved in the pathogenesis of abdominal aortic aneurysms. In this review, we provide an overview of the role of iron overload in AAA progression and explore its potential pathological mechanisms. Although the exact molecular mechanisms of iron overload in the development of AAA remain to be elucidated, the inhibition of iron deposition may offer a promising strategy for preventing these aneurysms.

## 1. Introduction

Abdominal aortic aneurysm (AAA) is a chronic vascular degenerative disease characterized by progressive segmental dilation of the abdominal aorta [1]. This condition results from the destruction of the extracellular matrix (ECM) and weakening of the arterial wall, which is often due to intraluminal thrombus formation [1]. Additionally, AAA is particularly prevalent in populations aged >65 years, and its incidence has markedly increased over the past decade. Although most AAA are asymptomatic, they are one of the leading causes of death in cardiovascular diseases due to rupture risk [1]. Aortic rupture caused by progressive dilation of the aortic wall is particularly catastrophic, with a high mortality rate reaching 80% [1]. In the United States, approximately 15,000 deaths are attributed to ruptured AAA annually [2]. Despite its severe implications, the underlying pathogenesis of AAA formation and rupture have not been fully elucidated.

While surgery is the definitive strategy for preventing AAA rupture [1], it provides no therapeutic advantage to patients with small AAAs or surgical contraindications. Despite numerous experimental and clinical studies aiming to develop effective medical therapies for AAA, no pharmaceutical treatment is currently available to arrest or limit AAA growth or to prevent aortic rupture [3]. Therefore, the management of AAA remains clinically challenging. Developing an effective therapy to prevent AAA progression and identify biological markers capable of predicting the risk of rupture in AAA is urgently needed.

Iron is a crucial micronutrient and an essential element for numerous biological and metabolic processes [4, 5, 6, 7]. In humans, iron is involved in erythropoiesis, oxygen transport and storage, mitochondrial energy metabolism, and deoxynucleotide and lipid synthesis [4]. The maintenance of iron homeostasis is crucial for the survival and function of cells and organisms, and disturbed iron homeostasis can result in tissue damage and harm to the body [5, 6, 7]. A series of epidemiological studies have indicated that both iron deficiency and overload can be detrimental to the human body and are associated with adverse clinical outcomes, particularly an increased risk of cardiovascular events [5, 6, 7]. Iron deficiency and overload promote the progression of many cardiovascular diseases, such as atherosclerosis, heart failure, and hypertension [5, 6, 7]. Recent evidence has indicated that iron overload is involved in the pathogenesis of AAA. In this review, we discuss the role of iron overload in AAA progression and examine potential mechanisms involved in its pathogenesis.

## 2. Iron Metabolism and Iron Overload

Iron levels in the body are regulated through a balance between iron absorption, storage, loss, and mobilization [8]. Postnatal iron content and distribution in the body are mainly determined by four key cell types: duodenal enterocytes that absorb dietary iron, erythroid precursors that affect iron utilization, reticuloendothelial macrophages that regulate iron storage and recycling, and hepatocytes that affect endocrine regulation and iron storage [9]. In the body, iron is distributed in tissues through blood plasma and erythrocyte hemoglobin is the main iron pool [4]. Under physiological conditions, communication between iron uptake and consumption from iron stores is tightly controlled to maintain metabolism in the body.

The iron status biomarkers included serum iron, transferrin, transferrin saturation, and ferritin levels [4, 5, 10, 11]. Serum iron levels are negatively regulated by hepcidin, a peptide hormone synthesized in the liver [10]. Hepcidin binds to the iron export protein ferroprotein and promotes its internalization and degradation while suppressing ferroprotein-mediated dietary iron absorption and transport into the circulatory system, thus resulting in the accumulation of iron [12]. Hepcidin secretion is regulated by extracellular iron levels, during iron-deficient conditions, hepcidin is downregulated, and ferroprotein-mediated iron export from the duodenal mucosal cells and the transfer of iron to transferrin can proceed unimpeded [10]. Meanwhile, hepcidin secretion increases in the presence of excess iron [10]. Transferrin is mainly synthesized in the liver and plays a central physiological role in iron transport at the sites of absorption, storage, and utilization [11]. Iron is bound to the iron-transport protein transferrin and is transferred to tissues through blood plasma [4]. In humans, serum transferrin levels are regulated by iron concentration; they are upregulated and downregulated during iron deficient and overload conditions, respectively [11]. In addition, transferrin saturation reflects the amount of iron bound to circulating transferrin, while ferritin is responsible for managing iron storage within various cell types [5]. Both transferrin saturation and serum ferritin levels are important biomarkers of iron stores and play a central role in maintaining iron homeostasis [5]. Clinically, these markers are used to assess the severity of iron overload [5]. A notably high transferrin saturation level suggests iron overload [10].

Under normal physiological conditions, iron homeostasis remains stable. However, abnormal iron metabolism occurs when its balance of iron metabolism is disturbed under pathological conditions. These disorders range from iron deficiency to iron overload and are widespread health problems worldwide. Iron overload is the excessive accumulation of iron storage, reflecting dysfunction of iron status or erythroid signal in the body [9]. This imbalance in intracellular iron homeostasis has a toxic effect that is detrimental to most cells and organs [13]. Iron accumulation in different organs leads to different clinical complications, including those associated with carcinogenesis [13]. Iron overload, a prevalent disorder of iron metabolism, affects many populations, particularly the elderly and patients with chronic diseases [1, 5]. The reasons for iron overload are often multifactorial, mainly caused by hereditary hemochromatosis, abnormal dietary iron absorption, parenteral administration, and drug-induced and long-term use of blood transfusions [9, 10]. In addition, iron overload is classified into two main types: primary and secondary. Primary iron overload results from hereditary hemochromatosis, a congenital disturbance in iron metabolism that affects approximately 1% of the population [8, 14]. In contrast, secondary iron overload is primarily caused by other disorders that predominantly present with cardiac, hepatic, and endocrine manifestations [14].

## 3. Relevance of Iron Overload in AAA

Iron overload is involved in many cardiovascular diseases, contributing to their onset and progression. Mortality due to secondary iron overload predominantly stems from cardiac complications including myocardial infarction, congestive heart failure, and arrhythmias [14]. Previous studies, both animal and human, have identified iron accumulation in atherosclerotic lesions [15, 16]. Iron accumulation occurs at the onset of atherosclerotic plaque formation, and increased iron storage is linked to an increased risk of atherosclerotic events, whereas iron depletion may protect against atherosclerosis progression [15, 17]. Additionally, the heart is particularly susceptible to damage caused by iron overload. Excessive iron accumulation in the heart directly damages cardiomyocytes, resulting in myocardial injury and cardiac dysfunction, particularly after ischemia/reperfusion [18, 19].

**Fig. 1.**
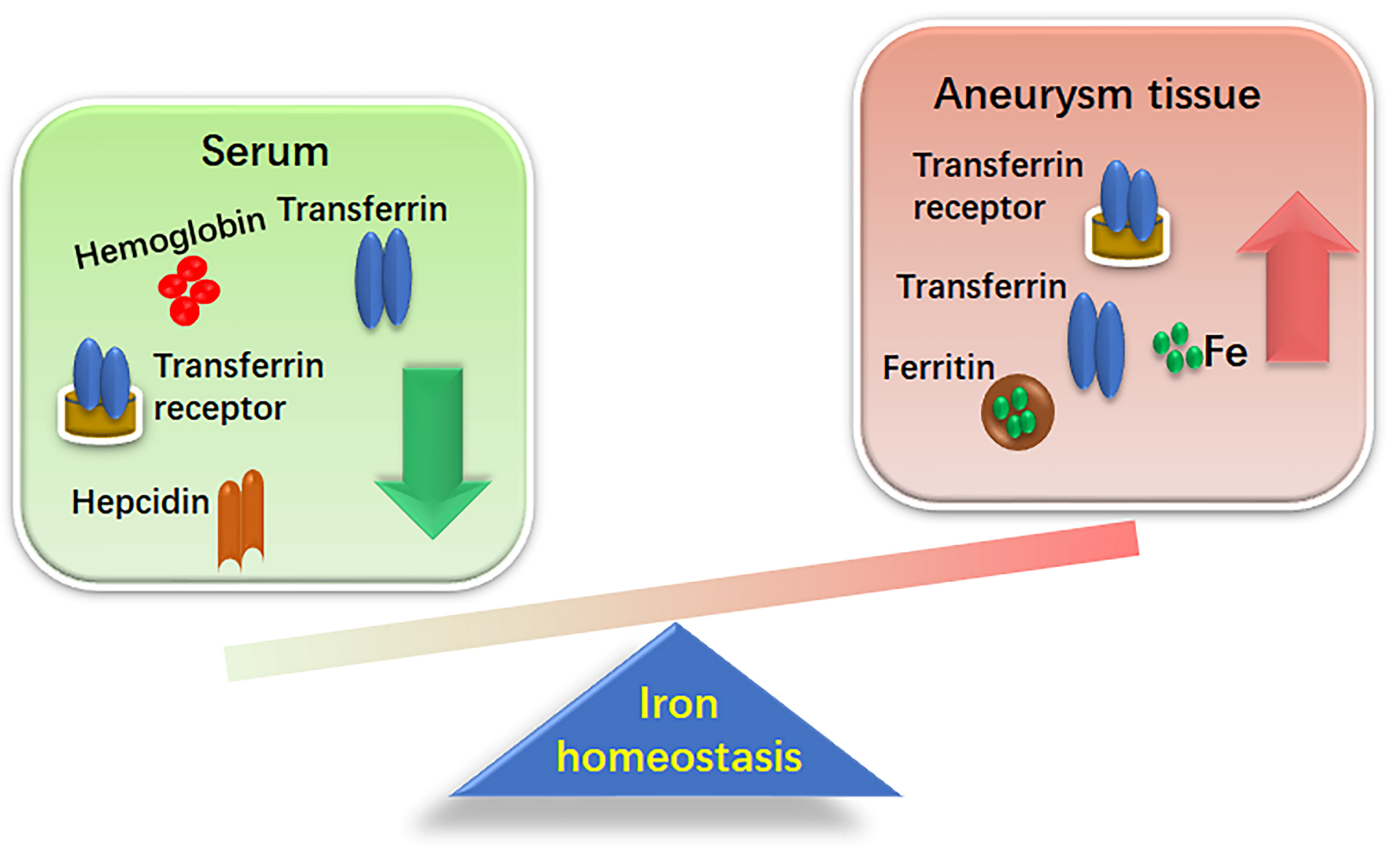
**Differential iron distribution between aneurismal tissues and serum**. This figure demonstrates the altered iron homeostasis in the context of aneurysmal disease. This disparity highlights a shift in iron dynamics from circulating serum to tissue deposition, potentially contributing to the pathophysiology of aneurysm progression.

Recent studies have shown an association between AAA and systemic iron metabolism as depicted in Fig. 1. Increased iron retention in aneurysmal tissues and decreased serum iron levels have been observed in both patients with AAA and animal models [20, 21]. A study by Sawada *et al*. [21], analyzed human aortic walls collected during surgery and compared them to aortic walls from a mouse model of AAA, induced by angiotensin II (Ang II) infusion in apolipoprotein E-knockout (*ApoE*^-⁣/-^) mice. Significant iron accumulation was observed in both human and murine AAA walls, as assessed by Berlin blue staining [21]. Moreover, tissue homogenates revealed that the aortic iron content of was significantly higher in AAA walls than in non-AAA walls [21]. A separate prospective clinical study examined eighty male hospitalized patients who underwent surgery for AAA or aortic occlusive disease (AOD) [22]. The results showed that iron expression levels were significantly higher in aneurysmal tissues compared to the aortic tissues of the AOD group [22]. Martinez-Pinna *et al*. [20] reported significant findings regarding iron metabolism in AAA patients: when compared to controls, these patients had considerably lower levels of circulating iron, transferrin, and hemoglobin concentrations, while their hepcidin levels were significantly higher. Moreover, serum levels of iron, transferrin, and hemoglobin were negatively correlated, while hepcidin was positively correlated with aortic diameter in patients with AAA [20]. Immunohistochemistry and western blot analysis of 10 AAA tissue samples collected during surgery showed that the protein expression of iron metabolism parameters (transferrin, transferrin receptor, and ferritin) was significantly increased in AAA tissues [20]. These findings suggest that AAA is associated with localized iron retention and disrupted iron recycling, which correlates with a decreased hemoglobin concentration [20]. As the main iron pool, reduced hemoglobin concentration was independently associated with the presence of AAAs and its clinical outcomes [20]. In contrast, a recent study presented a differing perspective on the role of hepcidin in AAA. They proposed that elevated hepcidin levels in smooth muscle cells (SMCs) of the aneurysm wall may be protective against AAA progression [23]. This finding introduces a new dimension to hepcidin’s potential as a disease-modifying agent and highlights the need to further experiments to assess its prognostic and therapeutic values beyond iron homeostasis disorders [23].

As an effector peptide for the renin-angiotensin system, Ang II has many significant biological properties, including vasoconstriction, aldosterone secretion, and blood pressure regulation [24]. Meanwhile, Ang II is associates with the inflammation response, ECM degradation, and vascular remodeling of the aorta [24]. Chronic Ang II infusion in *ApoE*^-⁣/-^ mice can successfully induce AAA formation, which is a widely accepted mouse model of AAA sharing many pathological similarities to human AAA [24, 25]. Several studies have indicated that chronic Ang II infusion in animals can alter iron distribution and induce iron accumulation in many organs, potentially influencing the function of the heart, liver, and kidneys [26, 27, 28]. Furthermore, Ang II also disturbs the balance of iron metabolism in vascular tissues, as Ang II administration promoted iron absorption and induced ferritin production and iron accumulation in the aorta [16, 26]. In a study by Ishizaka *et al*. [16], Ang-II-treated animals showed significant increases in both aortic ferritin protein expression and iron content, suggesting that Ang II induced iron deposition in the aortic wall. Supporting this notion, Tajima *et al*. [26] suggested that Ang II can influence iron metabolism by altering iron transporters, resulting in increased cellular and tissue iron content in mice. However, the mechanisms underlying iron deposition in AAA tissues have not been fully elucidated. Further studies are required to confirm this hypothesis.

## 4. Potential Role of Iron Overload in AAA

Accumulating evidence increasingly supports the role of iron metabolism and overload in AAA pathogenesis. It is now believed that iron accumulation may be a causal factor in AAA, rather than a mere consequence. This understanding could lead to improved management strategies for the disease. Iron overload is implicated in vascular remodeling under certain pathological conditions, including elevated Ang II levels. For instance, a study demonstrated that 14 days iron chelation therapy with deferoxamine significantly reduced the wall-to-lumen ratio and perivascular fibrosis area in Ang II-infused rats, suggesting that it mitigated Ang II-induced remodeling of the aortic wall [16]. Further research in an Ang II-induced AAA model in *ApoE*^-⁣/-^ mice found that an iron-restricted diet not only reduced AAA incidence dramatically—from 67% to 6%—but also completely prevented aortic rupture and resulted in smaller maximal abdominal aortic diameters compared to controls [21]. These results highlight iron’s crucial role in AAA progression. Notably, the benefits of iron restriction occurred independently of blood pressure changes, suggesting that the protective effects of iron limitation are specific to vascular pathology. Thus, dietary iron restriction or iron chelation could be promising strategies for preventing or treating AAA.

The role of iron overload in AAA development remains controversial. As a major intracellular iron storage protein, plasma ferritin concentration is regarded as a robust marker for assessing body iron stores [29, 30]. However, recent findings by Moxon *et al*. [29] challenge the established views. Their study found no significant differences in plasma ferritin concentrations between patients with or without AAA, and no correlation between ferritin levels and aortic diameter [29]. Additionally, during the 12–36 months follow-up period, the results indicated no significant difference in the rate of AAA diameter growth in patients regardless of iron overload [29]. These results led them to speculate that iron overload may not play a critical role in AAA pathogenesis and questioned the efficacy of iron reduction as a therapeutic strategy [29]. Given the conflicting evidences on the role of iron overload in AAA progression, further investigations are warranted.

## 5. Potential Mechanisms of Iron Overload in the Development of AAA

### 5.1 Iron Overload: Promoting Inflammation, Matrix Metalloproteinase Production, and Influencing Macrophage Phenotypes

The mechanisms underlying iron deposition in AAA tissues are not completely understood. It is well known that chronic inflammation, oxidative stress, excessive matrix degradation and vascular smooth muscle cell apoptosis are critical factors in AAA histopathology [31]. However, vascular inflammation also plays a central role in AAA onset and progression [32]. As the main proinflammatory cells, activated macrophages secrete inflammatory cytokines and produce proteolytic enzymes, such as matrix metalloproteinases (MMPs), that act in concert to progressively degrade wall elastin and mediate tissue destruction, all of which can promote AAA growth and rupture [1, 32]. In Ang II-infused AAA mouse models, macrophage accumulation was observed in the adventitia of the aorta [25, 33]. The recruitment and infiltration of macrophages into the arterial wall involves vascular remodeling and is a prominent feature of AAA progression [1, 34]. Inhibition of macrophage accumulation and blunted local inflammation may protect against AAA development in animal models [25, 32].

Macrophages are considered safe repositories of stored iron and play a central role in iron homeostasis [15]. Prior studies have indicated that iron storage by macrophages within atherosclerotic plaques played a central role in the progression of atherosclerosis, and that iron overload in macrophages promoted the progression of atherosclerosis [35, 36]. In a study by Kitagawa *et al*. [37], *ApoE*^-⁣/-^ mice were administered Ang II or saline (control group) to induce AAA. A combination of Perl’s iron staining and immunohistochemistry showed accumulation of iron and macrophages in AAA tissue [37]. Importantly, iron staining co-localized with macrophage infiltration [37]. In contrast, minimal Perl’s iron staining and macrophage expression were observed in the suprarenal aortic tissue of control mice [37]. Consistent with these findings, Turner *et al*. [38] revealed that the areas in which iron accumulated corresponded to macrophage-infiltrated areas in AAA tissues. These results suggested an inherent association between iron stores and macrophage accumulation during AAA progression.

Evidence has shown that high intracellular iron concentrations in macrophages rather than increased systemic iron levels drive inflammation [39]. Higher levels of intracellular iron in macrophages can promote the production of pro-inflammatory cytokines and reduce nitric oxide synthase activity [36]. Valenti *et al*. [40] showed that macrophage iron levels were positively correlated with the release of monocyte chemoattractant protein-1 (MCP-1) in high-risk individuals. In addition, inflammatory mediators can induce ferritin expression. In the *in vitro* experiments, tumor necrosis factor-alpha (TNF-α) specifically induced the synthesis of the ferritin H subunit in fibroblasts and other cells [41]. Decreased intracellular iron content plays a beneficial role in suppressing macrophage accumulation and blunting inflammatory responses. A previous study showed that dietary iron restriction attenuated the infiltration of macrophages and the expression of inflammatory- and fibrotic-related genes, including MCP-1, interleukin-6 (IL-6), transforming growth factor-β (TGF-β), collagen I and III in aortic walls in Ang II-infused mice, suggesting iron restriction can inhibit inflammation and fibrotic response induced by Ang II in AAA mice [21]. Endothelial nitric oxide synthase protects vascular cells from oxidative damage and its uncoupling contributes to severe vascular remodeling and leads to the predisposition of AAA formation [42]. Therefore, a deficiency in nitric oxide synthase activity caused by increased intracellular iron levels also accelerates the development of AAA.

Macrophages are the primary sources of MMPs in human tissues. The accumulation of iron in macrophages further promotes MMP production. Notably MMPs, particularly MMP-2 and MMP-9, are the predominant proteinases in the aortic wall, contribute to ECM degradation and vascular remodeling, and play a central role in AAA development [43]. However, MMP deficiency suppressed aneurysm formation in mouse models [43]. Recently, iron overload was reported to be involved in MMP activity and c-Jun N-terminal kinase (JNK) phosphorylation in AAA animal models. Sawada *et al*. [21] showed that the activity of MMP-2 and MMP-9 and the phosphorylation of JNK were significantly increased in the aortas of Ang II-induced AAA mouse models, whereas these increases were suppressed in mice that received an iron-restricted diet. In another in vivo experiment involving *ApoE*^-⁣/-^ mice, a three-month low-iron diet appeared to reduce iron deposition and MMP-9 expression while significantly increasing collagen content in lesions, suggesting that dietary iron restriction preserves lower matrix integrity [44]. Further *in vitro* experiments support this notion, showing that Ang II upregulated MMP-9 activity and JNK phosphorylation in macrophages, effects that were reversed by iron chelation treatment with deferoxamine (DFO) [21]. Increasing the concentration of ferric ammonium citrate was aloes found to upregulate MMP-9 expression in murine macrophages [44]. These results suggest that reducing the accumulation of iron in macrophages may protect against AAA progression.

Macrophages can be broadly classified into two subtypes: M1 and M2. The M1 macrophages are major players in inflammation, producing pro-inflammatory cytokines and inducing the secretion of oxidants, contributing to tissue degradation [45]. Conversely, M2 macrophages are involved in anti-inflammatory responses [45]. In AAA tissues, a predominance of M1 over M2 macrophages is observed in the adventitial layer of the aortic wall, where more extensive degradation occurs [31]. The phenotype of macrophages is influenced by their iron metabolism status [46]. Macrophage subtypes exhibit differences in iron management [35, 46]. Particularly, M1 macrophages sequester iron by downregulating ferroprotein and upregulating ferritin, which increases iron storage and limits iron discharge. In contrast to M1 cells, M2 macrophages have an iron-release phenotype with a higher capacity for heme uptake and non-heme iron release, with high levels of ferroprotein and low levels of ferritin [34, 36, 46]. Thus, M2 macrophages exhibit low iron levels by increasing iron excretion and limiting iron storage [36]. Under inflammatory conditions, iron accumulation in macrophages drives them toward the pro-inflammatory M1 phenotype, which may gradually increase the local inflammatory response and accelerates AAA development [46]. In contrast, an iron-restricted diet or iron chelation treatment can induce differentiation of macrophages into the M2 phenotype [36].

### 5.2 Iron Overload Induced the Dysfunction of Vascular Endothelial Cells

Endothelial cells play a central role in maintaining vascular homeostasis. Vascular endothelial cell dysfunction is a critical early step in AAA progression, contributing to both inflammation and oxidative stress in the degenerating arterial walls [47, 48]. A prior study linked excess iron to endothelial dysfunction. Notably, iron deposits were found in endothelial cells within early atherosclerotic lesions, and redox-active iron was shown to mediate the inflammatory responses in these cells [49]. A clinical study involving 20 healthy volunteers demonstrated that intravenous iron supplementation impaired endothelium-dependent vasodilatation and acute endothelial dysfunction, confirming a direct link between excessive iron and endothelial impairment [50]. Similar detrimental effects were observed *in vitro*. Kamanna *et al*. [51] reported significant changes in human aortic endothelial cells after 4 hours of exposure to iron-sucrose, including a loss of normal morphological characteristics, cellular fragmentation, shrinkage, detachment, monolayer disruption, and nuclear condensation/fragmentation. Moreover, iron-sucrose treatment significantly impaired acetylcholine-mediated relaxation in phenylephrine-pre-contracted rat aortas [51]. In another study, human umbilical vein endothelial cells were cultured with 10 mM non-transferrin-bound iron [52]. The authors observed that both increased intracellular labile iron levels and endothelial dysfunction were accompanied by elevated levels of vascular cell adhesion molecule-1 (VCAM-1), intercellular adhesion molecule-1 (ICAM-1), and endothelial selectin levels [52]. These findings indicate a detrimental effect of iron accumulation on endothelial function, particularly under conditions where iron levels exceed the carrying capacity of transferrin, leading to non-transferrin-bound iron that activates and disrupts vascular endothelial cells [49].

Avoiding iron accumulation may play a beneficial role in improving endothelial function. Immunohistochemistry revealed that ferritin expression was markedly increased in aortic endothelial cells of Ang II-infused rats [16]. Iron chelator treatment decreases ferritin expression and attenuates vascular dysfunction induced by Ang II in the aorta [16]. The results suggested that lowering vascular iron stores through iron chelators can improve endothelial dysfunction [53]. Furthermore, a study by Zhang *et al*. [54] investigated the effects of the intracellular iron-chelator, DFO on TNF-α induced expression of adhesion molecules in human aortic endothelial cells. The results showed that DFO time- and dose-dependently inhibited the expression of E-selectin, VCAM-1, and ICAM-1 induced by TNF-α in human aortic endothelial cells [54]. In contrast, iron-saturated DFO did not affect the expression of these adhesion molecules [54]. Therefore, reducing iron overload may improve endothelial cell dysfunction, providing a possible protective mechanism against AAA formation and rupture.

### 5.3 Iron Overload Promotes Oxidative Stress

Several studies have shown that reactive oxygen species (ROS) and oxidative stress are involved in the pathogenesis of AAA [55]. Excessive ROS production and a heightened oxidative stress responses contribute to AAA development by promoting inflammation and facilitating ECM degradation and remodeling [39, 40]. In contrast, the inhibition of ROS production and oxidative stress has been shown to suppress aneurysm formation in mouse models [47, 56]. Labile iron, a powerful oxidant, induces oxidative stress by generating highly toxic hydroxyl radicals via the Fenton–Haber–Weiss reaction [57]. Increased iron levels catalyze free radical reactions, leading to the oxidation of low-density lipoprotein (LDL), which exacerbates oxidative stress, and subsequently leads to tissue damage [22]. A recent study by Sawada *et al*. [21] utilized 8-hydroxy-2^′^-deoxyguanosine (8-OHdG) staining to evaluate oxidative stress in human and murine aortic walls. The presence of 8-OHdG deposition in AAA walls was consistently associated with iron accumulation, as demonstrated by Berlin blue staining, and was positively correlated with the severity of iron deposition [21].

Human AAA is characterized by an intraluminal thrombus, which is rich in hemoglobin derived from erythrocytes [58]. Hemagglutination, the clumping together of red blood cells within the blood vessel, occurs in intraluminal thrombi and releases free hemoglobin, and consequently, free iron, which catalyzes the production of intracellular ROS and oxygen free radicals [59]. The oxidative activity in AAA is mainly associated with hemoglobin-related iron release from these intraluminal thrombi [58]. Once ROS production exceeds the capacity of cellular antioxidant systems, it triggers an oxidative stress response [60]. This process can cause extensive damage to lipids, proteins, nuclear DNA, transcription factors, and enzymes, leading to cell dysfunction, apoptosis, and ultimately damage to tissues and organs [60]. Iron overload exacerbates this process by intensifying oxidative stress, which plays a central role in the pathophysiology of AAA and the resultant tissue and organ injury [8].

In theory, reducing iron levels may effectively decrease iron-catalyzed oxidative stress and improve outcomes in diseases associated with oxidative stress. Studies have shown that dietary iron restriction effectively suppresses the progression of oxidative stress. In a study by Ikeda *et al*. [61], dietary iron restriction significantly decreased urinary albumin excretion and protected against diabetic nephropathy in *db/db* mice by reducing oxidative stress. Similarly, in mice with Ang II-induced AAA, dietary iron restriction significantly attenuated the extent of 8-OHdG-positive areas in the aortic wall, suggesting that oxidative stress was suppressed [21]. They hypothesized that the decreased incidence of AAA may be attributed to the suppressed oxidative stress [21]. Therefore, limited iron accumulation may attenuate oxidative stress, thus offering protection against AAA development.

### 5.4 Iron Load Induces Apoptosis of Vascular Smooth Muscle Cells

**Fig. 2.**
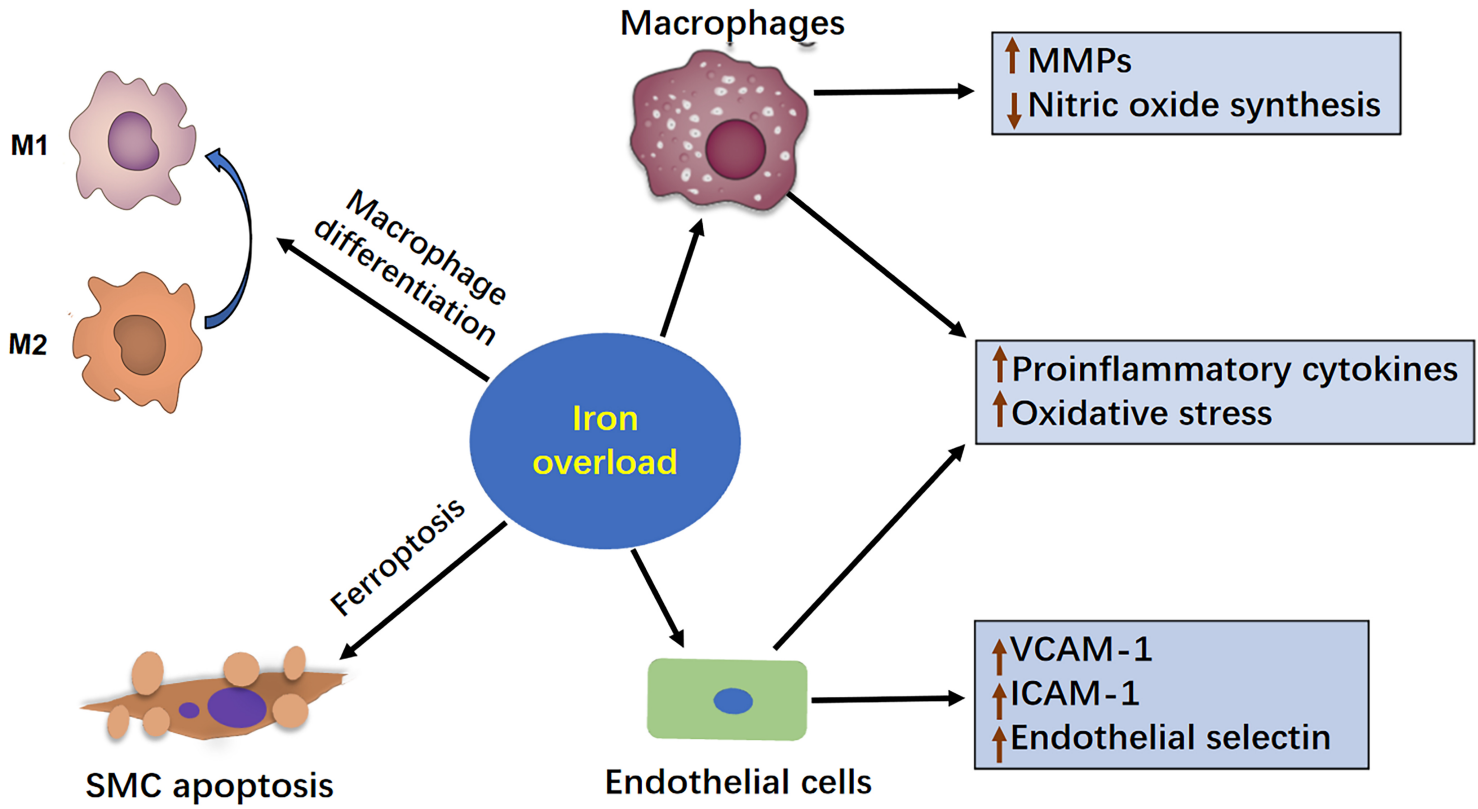
**The mechanism of iron overload in the development of AAA**. This figure illustrates how iron overload contributes to the development of AAA. Iron overload can exacerbate AAA progression by enhancing the inflammatory response, increasing MMP production, inducing oxidative stress, and ultimately promoting vascular apoptosis in SMCs. Therefore, maintaining a balanced intracellular iron level is important for preventing AAA progression. AAA, abdominal aortic aneurysm; MMP, matrix metalloproteinase; SMC, smooth muscle cells; VCAM-A, vascular cell adhesion molecule-1; ICAM-1, intercellular adhesion molecule-1.

Vascular smooth muscle cells (SMCs) play an important role in ECM metabolism. Chronic apoptosis of vascular SMCs induces degradation of the ECM and lumen dilatation, thereby increasing the susceptibility of the aneurysm to rupture [62, 63]. In contrast, preventing the loss of vascular SMCs prevents the progression of aneurysms [62]. Ferroptosis, a newly defined form of programmed cell death, is characterized by lipid peroxidation (Fig. 2) [36, 64]. Iron overload can exasperate lipid peroxidation via the Fenton reaction, inducing ferroptosis, and subsequently resulting in vascular SMCs loss [36, 64]. Ferroptosis occurs gradually during the progression of atherosclerosis [36]. Whether iron overload-induced ferroptosis also contributes to the pathophysiology of AAA still remains unclear.

## 6. Therapeutic Potential of Inhibiting Iron Deposition in AAA Treatment

Aberrant iron homeostasis in the body or vascular wall, influenced by altered vascular reactivity, contributes to atherosclerosis development [16]. Accumulating evidence implicates iron deposits as a crucial factor in atherosclerosis initiation, while restricting iron intake or inhibiting of iron accumulation may prevent progression of the disease [15, 16]. A recent study demonstrated that iron levels in newly formed atherosclerotic lesions in cholesterol-fed rabbits increased seven-fold compared to healthy arterial tissue, and that suppression of iron uptake delayed the onset of atherosclerosis [65]. To date, there are no known endogenous mechanism for the removal of excess iron from the body. Thus, limiting the systemic iron content or inhibiting excess iron in tissues remains the main approach for managing iron overload [8].

Iron chelation, specifically with deferiprone, is a primary method for reducing systemic iron levels and removing excess iron from the body. Matthews *et al*. [66] found that iron chelator treatment twice daily for 10 weeks significantly decreased the thoracic aortic cholesterol content and prevented the development of atherosclerosis in hypercholesterolemic rabbits. In another study by Minqin *et al*. [17], nine weeks of iron chelator treatment significantly reduced iron content in the lesions of cholesterol-fed rabbits, from 95 ppm dry weight to 58 ppm dry weight, as measured using nuclear microscopy. This treatment also significantly decreased the area of atherosclerotic lesions compared to controls [17]. These results suggest that iron inhibition can protect against atherogenic progression.

Atherosclerosis and AAA share many common risk factors, such as age, smoking, and hypertension, and exhibit similar pathological characteristics, such as inflammation, proteolysis, and apoptosis [67]. Inspired by the role of iron inhibition in atherosclerosis, exploring similar strategies for reducing iron accumulation may be beneficial to AAA therapy. Recently, One study has documented that iron restriction can suppress the formation of AAA in experimental animal models [21]. Based on the above results, the proposed mechanisms through which iron inhibition may combat AAA include a blunted inflammatory response, reduced oxidative stress, decreased MMPs production, lower matrix degradation, improved endothelial function, and reduced vascular SMCs apoptosis. Although promising results have been observed in both in vivo and in vitro experiments, further research is needed to determine whether dietary intervention or iron chelation treatment can effectively prevent AAA formation and rupture in patients with AAA.

## 7. Conclusions

While iron homeostasis is essential for health, both systemic and localized iron disruptions to homeostasis have been observed in patients with AAA. Iron overload is involved in the pathology of AAA, making its early identification and correction a priority for potential clinical benefits. Currently, the management of iron overload depends mainly on proper dietary restrictions and iron chelation therapy. Although iron chelators can reduce the iron burden, concerns remain regarding the side effects from their long-term use. Therefore, the development of safer and more effective pharmacotherapies to prevent iron overload represents a promising approach for preventing or treating AAA. Future systemic studies are needed to clearly establish the link between iron overload and AAA, and to elucidate the specific effects of iron overload on the AAA process. A deeper understanding of the role of iron in AAA could pave the way for novel interventions to target the pathological effects of excess iron.

## Abbreviations

AAA, abdominal aortic aneurysm; Ang II, angiotensin II; AOD, aortic occlusive disease; ApoE^-⁣/-^, apolipoprotein E-knockout; DFO, deferoxamine; ECM, extracellular matrix; ICAM-1, intercellular adhesion molecule-1; IL-6, interleukin-6; JNK, c-Jun N-terminal kinase; LDL, low-density lipoprotein; MCP-1, monocyte chemoattractant protein-1; MMP, matrix metalloproteinase; ROS, reactive oxygen species; SMCs, smooth muscle cells; TGF-β, transforming growth factor-β; TNF-α, tumor necrosis factor-alpha; VCAM-1, vascular cell adhesion molecule-1; 8-OHdG, 8-hydroxy-2^′^-deoxyguanosine.
